# Race and indigeneity in human microbiome science: microbiomisation and the historiality of otherness

**DOI:** 10.1007/s40656-024-00614-w

**Published:** 2024-04-02

**Authors:** Andrea Núñez Casal

**Affiliations:** 1 Department of Philosophy and Anthropology, Universidad de Santiago de Compostela, Santiago de Compostela, Spain; 2 Department of Science, Technology, and Society, Institute of Philosophy, Spanish National Research Council (IFS-CSIC), Madrid, Spain

**Keywords:** Human microbiome, Race, Indigeneity, Bioprospection, Personalised medicine, Microbiomisation, Historiality

## Abstract

This article reformulates Stephan Helmreich´s the ¨microbiomisation of race¨ as the historiality of otherness in the foundations of human microbiome science. Through the lens of my ethnographic fieldwork of a transnational community of microbiome scientists that conducted a landmark human microbiome research on indigenous microbes and its affiliated and first personalised microbiome initiative, the American Gut Project, I follow and trace the key actors, experimental systems and onto-epistemic claims in the emergence of human microbiome science a decade ago. In doing so, I show the links between the reinscription of race, comparative research on the microbial genetic variation of human populations and the remining of bioprospected data for personalised medicine. In these unpredictable research movements, the microbiome of non-Western peoples and territories is much more than a side project or a specific approach within the field: it constitutes the nucleus of its experimental system, opening towards subsequent and cumulative research processes and knowledge production in human microbiome science. The article demonstrates that while human microbiome science is articulated upon the microbial ‘makeup’ of non-wester(nised) communities, societies, and locales, its results and therapeutics are only applicable to medical conditions affecting rich nations (i.e., inflammatory, autoimmune, and metabolic diseases). My reformulation of ¨microbiomisation of race¨ as the condition of possibility of human microbiome science reveals that its individual dimension is sustained by microbial DNA data from human populations through bioprospecting practices and gains meaning through personalised medicine initiatives, informal online networks of pseudoscientific and commodified microbial-related evidence.

**Supplementary Information:**

The online version contains supplementary material available at 10.1007/s40656-024-00614-w.

## Introduction

Back in the late 1960s, microbiologist Lynn Margulis proposed a symbiotic vision of life with her endosymbiotic theory of evolution, also known as symbiogenesis `a theory of coming together, of merging cells of different histories and abilities’ (1999, p. 40). As she recalls in her book *The symbiotic planet* (1999), symbiogenesis started attracting scientific attention during the 1970s and 1980s, when studies in genetics and molecular biology confirmed prokaryotic cells (such as bacteria) led to eukaryotic cells (such as human cells). In a visionary statement, Margulis alluded to human-microbial entanglements as: ‘we are walking communities’, she explained, ‘ten percent or more of our body weight is bacterial [in its evolutionary origins, ANC], and it is just foolish to ignore that’ (Mann, 1991, p. 378). Now, three decades later, these very same words are commonplace as biological and biomedical studies of human microbiome science highlight the symbiotic and coevolutionary history between microbes and humans.

The human microbiome, that is, the collective genomes of microorganism that populates the different organs, cavities, and surfaces of the human body, has gained an unprecedented importance in a renewed biomedical and popular understanding health and disease in the twentieth-first century. This fast-moving area of biomedicine has challenged the tenet of a fixed and self-contained human nature by recognising the role of microbes along with environmental and lifestyle factors in the shaping of the immune function. Most microbes inhabiting the interior, surfaces, and orifices of the human body are symbiotic and commensal organisms, essential for metabolic, immunological, and even behavioural functions (Blaser, 2006).

Onto-epistemic debates around cooperative systems of cells in the light of the human microbe have resulted in a redefinition proposal of the unit of natural selection as the holobiont, which entangles the classic category of the organism and its associated microbial communities (Dupré & O´Malley, 2007). The promises around multispecies co-evolutionary processes and living futures encapsulated by the repeated motto that we, humans, ‘are 99 per cent microbial’ has led to some celebratory and preliminary claims and analyses in the philosophical and social studies of the human microbiome, particularly in terms of the inauguration of a new scientific area away from anthropocentrism (Benezra et al., 2012; Cohen, 2009; Dupré, 2012; Hird, 2009). Some authors have argued that human microbiome research offers ‘a more profound view of our humanness´—transforming our categories of “community”, “individual”, and “life”’ (Benezra et al., 2012, p. 6380). Over the past decade, as multispecies sensitivities have increasingly become a very prolific and eclectic theme in the social sciences and humanities, critical analysis of microbial biosocieties have incorporated a wide variety of topics (Cañada et al., 2022; de Lima Hutchison, 2022; Giraldo Herrera, 2018; Greenhough, 2012; Haraway, 2008, 2016; Helmreich, 2009, 2016; Hinchliffe, 2015; Kirksey & Helmreich, 2010; Kohn, 2007; Lorimer, 2016; Nading, 2014, 2016; Paxson, 2008; Santesmases, 2018; Tsing, 2015; Yates-Doerr, 2015), including human-microbial entanglements in public health systems (Nading, 2014), new therapies with fecal transplantation (Beck, 2021; Lorimer, 2016), feminist embodied methods and live experience mapping (Núñez Casal, 2019, 2021a, 2021b) and microbes as new bioeconomic agents (Delgado, 2021), to name a few.

As part of this heterogenous body of critical microbial science literature, Stefan Helmreich has introduced the neologism of the ¨microbiomisation of race¨ (2016, p. 67) as a framework for the re-instantiation of race through microbial genomics. Drawing on Helmreich ´s argument, Amber Benezra has recently referred to race in microbiome science as a ¨ghost variable¨, in *absent presence* (M’charek et al., 2014), and thus, characterised by its slipperiness and conflation with terms like ¨nationality¨ or ¨geographical ancestry¨ (Benezra, 2020; see also de Lima Hutchison & Núñez Casal, 2023). In this line, as I have shown elsewhere, the processes of microbiomisation involve the scientific production of molecularised, unidimensional, and essentialist social categories of difference (including race, but also gender, class) through the characterisation and classification of microbial diversity (Núñez Casal, 2019, 2021a, 2021b, 2024).

Contributing to these perspectives that critically situate race, particularly *race as indigeneity*, in the human microbiome yet going a step further, beyond race as a ¨ghostly variable¨ or a retrogressive meaning, byproduct of human microbiome scientific production, this article argues that race *is* the foundation of microbial science´s ¨experimental systems¨.^1^ Here I draw from Hans-Jörg Rheinberger´s concept of ¨historiality¨, that is, the historical structure of scientific action that ¨escapes the classical notions of linear causation, retroaction, influence, and dominance (…) ¨ (1994, p. 69). The historiality of otherness in microbial science involves ¨remnants of older narratives¨, specifically the reinscription of race science in postgenomic diversity research (Fullwilley, 2007) as well as ¨fragments of narratives that have not yet been told¨ (Rheinberger, 1994, p. 77), that is, race as the scaffolding upon which human microbiome science´s experimental system rests**.**

The article reformulates Stephan Helmreich´s the ¨microbiomisation of race¨ as the historiality of otherness in the foundation of microbial science by drawing on ethnographic fieldwork of a transnational community of microbiome scientists that conducted a landmark human microbiome research in the Peruvian and Brazilian Amazon (2012–2014), the ¨Microbiomes of Homes across Cultures¨ (MHC) project, and of its associated and first personalised microbiome initiative, the American Gut Project (AGP). Through the lens of this particular microbiome study and its associated personalised medicine initiatives along with empirical data from interviews with microbiome scientists, attendance of microbiome conferences, and an analysis of scientific publications, I follow and trace this influential western community of scientists, their research collaborations and initiatives, their research processes and lines of argument, all key actors and actants in the emergence of human microbiome science a decade ago. In doing so, I show the links between the reinscription of race, comparative research on the microbial genetic variation of human populations and the remining and commodification of individualised microbial profiles. In these unpredictable research movements, the microbiome of ‘uncontacted peoples’, such as the high Orinoco Yanomamis of Venezuela or the Peruvian Amerindians of Checherta, became the foundations microbiome science. Here, the microbiome of non-Western peoples and territories is not a side project or a specific ‘approach’ within the field but, rather, constitutes the nucleus of its experimental system, opening towards subsequent and cumulative experimental and knowledge production in the field. What the spontaneous and unforeseen recurrence of race in microbial postgenomics reveals is that an ¨experimental system has *more stories* to tell than the experimenter at a given moment is trying to tell with it¨ (Rheinberger, 1994, p. 77).

I divided the article into two main blocks or sections. In the first part, I provide the methodological and empirical details of the US human microbiome study I documented and analised between 2013 and 2018. I first outline a genealogy of ‘transculturation’, an intriguing key concept of the MHC research, often interchangeable with ‘westernisation’ and ‘urbanisation’. I argue that these sociologically rooted concepts embody and establish the basis of ¨microbiomisation ¨ as the processes by which human microbiome science takes social groups as pre-existing, ‘natural’ phenomena and biologises them by creating microbes and microbial profiles and attributing these to them. Engaging with science studies literature on race (El-Haj, 2007; TallBear, 2013; Wade et al., 2014), I demonstrate that the experimental design of MHC and similar human microbiome projects starts from non-scientific assumptions about cultural and social differences in populations. This first part of the article provides the necessary background to reformulate the notion of the “microbiomisation of race” as the foundation and condition of possibility of human microbiome science in the second part.

The second section zooms in on how the ¨microbiomisation of race¨ is enacted (i.e. practised) through bioprospecting microbial biodiversity from non-Western humans, non-humans and more-than-human populations and territories. Importantly, the MHC project has several online and offline ramifications. I follow those online ramifications and examine the para-ethnographic evidence (non-scientific) of the microbiome online community associated with the American Gut Project (AGP), the first personalised microbiome initiative. Drawing on empirical data from the AGP and affiliated online microbiome initiatives and material from interviews, this section of the article shows how the ¨microbiomisation of race¨ is co-constituted by two interlocking processes or phases: (1) the bioprospection of non-Western human and more-than-human microbial biodiversity and (2) the ¨remining¨ (Neimark & Wilson, 2015) of bioprospected data for personalised microbiome profiling online platforms and initiatives.

My argument is that while human microbiome science is articulated upon the microbial ‘makeup’ of non-Wester(nised) communities, societies, and locales, its results and therapeutics— that is, the health contributions of this biomedical area—are only applicable to medical conditions affecting rich nations (i.e., inflammatory, autoimmune, and metabolic diseases). This movement reveals that the individual dimension of human microbiome science is sustained by microbial DNA data from human populations through bioprospecting practices and gains meaning through personalised medicine initiatives, informal online networks of pseudoscientific and commodified microbial-related evidence.

## Microbes and transculturation: glimpses from a conference

The word ‘transculturation’ from microbial ecologist María Gloria Dominguez-Bello’s abstract title ‘Genomics and global health in the context of transculturation’ first caught my attention at the conference ‘Infectious Disease Genomics and Global Health’ (2013), organised by the Wellcome Trust between the 16th and the 18th of October 2013 at the Wellcome Trust Genome Campus at Cambridge (UK). This intriguing word, I learnt at the conference, was an analytical tool to describe the degree of westernisation, from ‘unimpacted peoples’ to communities adopting a western lifestyle (Dominguez-Bello, 2013). I found myself immersed in an ambiguous lexical world in which ‘scientific’ words such as microbiota, microbiome, resistome, and antibiotic- resistant genes (AR) were tangled up with anthropological categories such as ‘transculturation’, ‘unimpacted peoples’, ‘westernisation’, ‘modern practices’ and ‘globalisation’. Was the use of such idiosyncratic rhetoric evidencing something novel in the life sciences, particularly in relation to the traffic between nature and culture?

I traced the genealogy of the word ‘transculturation’ back to the work of anthropologist Fernando Ortiz in his book *Cuban counterpoint: Tobacco and sugar (1995).* In the aftermath of Spanish colonialism in Cuba, Ortiz suggests the term ‘transculturation’ to refer to the converging of two cultures and the creation of a new one (neo-culturation), in contrast to the unidirectional acquisition of another culture (acculturation). Was the term ‘transculturation’ an occult reference to the anthropologist Fernando Ortiz?; Could microbes be compared to sugar and tobacco and thus be seen through the lens of Ortiz, as both non-human social actors and commodities transforming the collective identities and social history of contemporary societies?; Could Dominguez-Bello be considered as a representative of a new way of approaching biological questions in which culture and emancipation (as a non-Western woman scientist) go in hand?

Following the Wellcome Trust conference in October 2013, I established initial contact with Dominguez-Bello, expressing my interest in conducting ethnographic fieldwork on her microbiome research. Soon after, she invited me to take part in the next expedition she and her research team were organising to Manaus (Brazil), between 8 and 19 December 2013. There, they would be conducting microbial DNA sampling of surfaces, house objects, and the skin of humans and non-human animal inhabitants of ten modern apartments. The DNA data gathering that Dominguez-Bello’s research team would perform in Manaus is part of a larger innovative research project entitled ‘Microbiomes of Homes across Cultures’ (MHC), funded by the US-based Sloan Foundation Programme ‘Microbiology of the Built Environment’. Sadly, due to administrative constrains, I could not join the scientific team in Manaus. I was lucky that Dominguez-Bello proposed to me, as an alternative, to visit her laboratory at the University of Puerto Rico, Río Piedras Campus (UPR-RP), in San Juan and at the New York Langone Medical Centre, in New York soon after her and her team came back from Manaus.

In what follows, I reflect on a research project that stretched from September 2013–2018. The findings discussed map the emergence of the western area of human microbiome research through the lens of the landmark US ¨Microbiomes of Homes across Culture¨ (MHC) project and its associated community of collaborators and affiliate initiatives. The core ethnographic study consisted in a five-week visit to Dominguez-Bello´s lab at the UPR-RP and the NYU between December 2013 and February 2014. There, I familiarize myself with her team, their research area, methods, and instruments. I conducted in-depth and semi-structured sixty-minute, one-to-one interviews with Dominguez-Bello, all her team consisting of four doctoral and one postdoctoral researcher, two architects and one anthropologists along with interviews with collaborators including prominent NYU microbiologists Martin J. Blaser, Zhiheng Pei, Daniel Littman and, in the UK, with multifaceted geneticist, physician, and popular science writer on microbiome science Tim Spector (King´s College London) and renowned microbiologist and immunologist Graham Rook (University College London). All participants agreed and signed the informed consent form, and all the interviews were recorded digitally and transcribed verbatim. The interviews I conducted in Puerto Rico were in Spanish, while the ones conducted in New York and London were in English.

Between July 2013 and June 2014, I attended six international conferences, including the first and successive ¨Exploring Human Host–Microbiome Interactions in Health and Disease¨ (2013), organised by the Wellcome Trust, at the Wellcome Trust Genome Campus, Hinxton, Cambridge (UK) and ¨The 1st London Microbiome Meeting¨ (2014) led by Tim Spector and organised by his Department of Twin Research, KCL.

Since 2013, I have also followed and documented the news and public statements made by this community of microbiome scientists in several online platforms. This mostly includes the American Gut Project (AGP) and, to a lesser extent, the Human Food Project (HFP), British Gut (BG), and Map My Gut (MMG), offshoot microbiome initiatives of the AGP.^2^ In addition, I have conducted participant observation of a six-week massive open online course (MOOC) offered by the research team leading the AGP at the University of Colorado Boulder.^3^

## Microbiomes of homes across cultures: the historiality of race in microbial science

At the Faculty of Biology of the University of Puerto Rico, Dominguez-Bello and doctoral student Jean Ruiz-Calderon tell me about the expedition they endured back in the summer of 2012. The first locality they visited was the hunter-gatherer community of Checherta (Peru), with approximately three hundred inhabitants. Checherta is an ‘isolated’ Achuar Amerindian community without drinking water or electric services, accessible only by taking an aeroplane to a jungle strip in Nuevo Andoas (Peru), plus a two-day boat trip towards the border with Ecuador. Arriving at the canoe port, locals, especially children, ran towards the team. The community had previously approved their visit through the mediation of a local interpreter and a catholic missionary, Father Luigi, who negotiated the details of the visit with the *Apu* (chief). But because this was a first-time visit, they had to wait several hours outside the village.

**Fig. 1:**
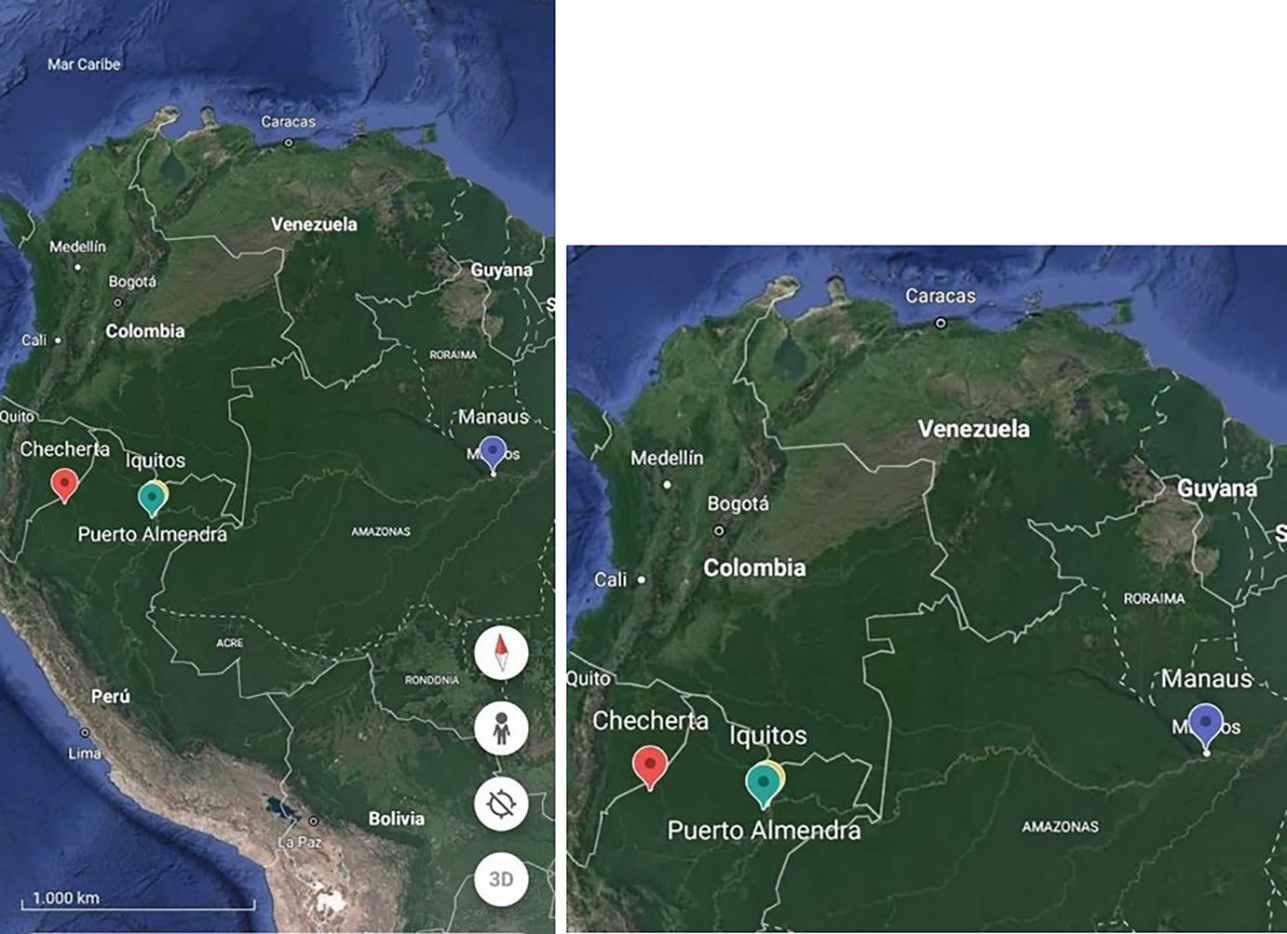
Google Earth still images showing the four locations at the same latitude of the Peruvian and Brazilian Amazon where the MHC team sampled microbial DNA. From West to East (in a gradient of transculturation—less to more westernised): huts of isolated Achuar villages (Checherta); rural settlement of an Amerindian-mestizo town (Puerto Almendros), mestizo cities (Iquitos), and mestizo modern buildings (Manaus)

The expedition is part of the US Sloan Foundation-funded “Microbiomes of Homes across Cultures” (MHC) project. A pioneer human microbiome study at that time, the project looked at changes in microbial patterns and changes across an evolution of lifestyle, from less to more “westernised” modes of living attending to urbanisation, diet and medicalisation among other sociocultural elements and characteristics of lifestyles. The scientific team consisted of microbial ecologists and other scientists coming from diverse fields including architecture, environmental engineering, and anthropology. They conducted two main expeditions (2012 – 2014) structured around four locations at the same latitude of the Peruvian and Brazilian Amazon (see Fig. 1) and sampled microbial DNA from the environment (air, surfaces of objects), human and non- human animal bodies.

The objective of the MHC study was to investigate whether the built environments (i.e. open-air huts) of Checherta—along with their inhabitants’ diet (non-processed food) and lack of exposure to antibiotics—correlate with a more diverse composition of their human microbiome as well as their environmental microbes. The contention of the PI of the project, Dominguez-Bello, was that ‘modern lifestyle has led to changes in microbial patterns in humans and their environments, with reduced microbial diversity … [producing] profound changes transmitted by descent, and perpetuated in future generations’ (personal correspondence, 2012). As she explained to me explains:

While our ancestors are no longer there, and studying their remains has important limitations, we can alternately study indigenous or African hunter-gatherers. These are cultures close to those of our ancestors, and we can compare them with urban lifestyles, because both are contemporary (M. G. Dominguez-Bello, personal communication, January 28, 2014)

Difference is co-produced with scientific lines of inquiry about microbial diversity. It is an indissociable element of microbial science´s experimental system, tangled up since its very inception.

Besides Western lifestyle practices and trends associated with diet, modes of delivery at birth (Dominguez-Bello et al., 2010a), or antibiotic usage (Bisgaard et al., 2011), the MHC project explored changes in the pattern and composition of microbiota associated with the quantity of time spent indoors and the lack of ‘natural’ ventilation of modern architectural designs (Dominguez-Bello, 2012, p. 3). In indoor environments, ‘humans are exposed to surfaces with a bacterial content that reflects the space and object uses’ and they ‘also shed microbes to the environment, and ventilation greatly affects microbial transmission by aerosol, which is of special interest in hospital design’ (p. 3) The study was part of an emerging interdisciplinary area in the of human–microbe entanglements in human constructed environments known as ‘microbiology (also “microbiomes”) of the built environment’. The field encompasses studies of microorganisms and various types of built environment, including houses, vehicles, hospitals, water systems, and clothing. Biologists collaborate with architects, designers, and doctors.

**Fig. 2:**
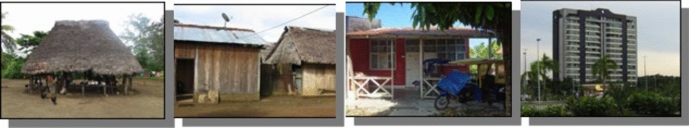
The four different types of housing architecture across the ‘transculturation gradient’ from left to right: Checherta (Peru), Puerto Almendra (Peru), Iquitos (Peru) and Manaus (Brasil). Courtesy of Jean Ruiz-Calderon

Prior to the expedition, Dominguez-Bello selected ten homes (or ‘huts’ in the case of Checherta) per four communities at the same latitude in the Amazon basin, with different degrees of urbanisation: Checherta (jungle), Puerto Almendras (rural), Iquitos (town), and Manaus (city) (see Fig. 2).

Once they got access to Checherta, Dominguez-Bello and Ruiz-Calderon tell me passionately, they took DNA samples from surfaces of objects, floors and walls, and humans (skin, nose, mouth, and anal swabs) and animals. After the team collected DNA samples with sterile cotton swabs, these were immediately stored in liquid nitrogen (− 80 °C) and kept frozen until shipping to Rob Knight Lab at the University of Colorado Boulder where collaborators extracted the DNA, as I will discuss later in the article. They repeated the sampling method in each of the four locations. The idea was that combining microbiological with architectural (e.g., room area, windows, and doors per room, sample height) and environmental measurements (relative humidity, temperature, light, air exchange rate, wind speed, ultrafine air particles, CO, CO_2_), the researchers can determine the composition and geography of microbes to establish comparisons between ¨indigenous¨ and ¨western microbes¨. The platonic metaphysis of dualisms between good and bad bacteria, between indigenous and industrialised/westernised microbiomes, figures as a key element of this microbiome science epistemic device and, crucially, of the design of its own internal experimental system.

### A genealogy of transculturation

In San Juan (Puerto Rico) in early 2014, I met Waleska Sanabria León, a biosocial anthropologist at the University of Puerto Rico. At that time, she had just become the anthropologist of the MHC project. She noted that acculturation was the preferred terminology in the first draft of the MHC Sloan Foundation proposal (W. Sanabria León, personal communication, January 28, 2015):

A set of cases will be selected from a continuum of settings that represent typical dwellings of the environments in a gradient of acculturation from isolated villages to cosmopolitan cities: isolated jungle communities/rural jungle settlements and small city/cosmopolitan city. We propose to choose villages in gradient of acculturation in Peru (Fig 1) and include a Latino community in Manhattan as the metropolis in the most acculturated end (Dominguez-Bello, 2012, p. 7).

In view of the negative connotation of ‘acculturation’ as a unidirectional process of cultural acquisition, Sanabria León proposed the alternative term ‘transculturation’. She clarified to me that the processes of fluidity and ‘non-localised quality’ of Appadurai’s work (1996) was an inspiration for her reformulation of acculturation as transculturation in the MHC research. Her point was to remark on the unsettled nature of cultures. The provenance of the reference for the use of transculturation was, and still is, unclear and cryptic (see also Casid, 2004).

Progressively, transculturation, westernisation, and urbanisation became interchangeable concepts in the MHC research, as the following quote reads:

While the world is converging toward Western lifestyles (a process known as transculturation/Westernization), there is a need to characterize the changes that occur during this convergence, and to provide insights into which factors may contribute to specific immunologic and metabolic diseases … (Ruiz-Calderon, 2015, pp. 19, 29).

In the journal article versions, however, transculturation no longer appears. Here, the experimental design and rationale of the MHC research is framed in terms of urbanisation, understood as an outcome of westernisation:

Westernization has propelled changes in urbanization and architecture, altering our exposure to the outdoor environment from that experienced during most of human evolution. These changes might affect the developmental exposure of infants to bacteria, immune development, and human microbiome diversity … This study addresses the associations between architectural design and the microbial biogeography of households across a gradient of urbanization in South America (Ruiz-Calderon et al., 2016, p. 1).

The progressive substitution of transculturation with the concepts of urbanisation and westernisation, I argue, elicits the socio-cultural and anthropological dimension of the MHC research, moving towards an architectural context under the framework of microbiology of the built environment. Talking about ‘urbanisation’, I suggest, sounds more technical and is less problematic in terms of research ethics (access to indigenous communities, sampling DNA, etc.). In other words, talking about buildings and design (i.e., urbanisation) instead of race, nation, and ethnicity (i.e., transculturation, westernisation) is a way to ‘sanitise’ scientific discourse, avoiding controversies and criticism.

The evolution of the concept of transculturation in the MHC research—how it travels, transforms, and ‘normalises’ itself as part of more common and accepted concepts in contemporary scientific discourse (i.e. ‘westernisation’, ‘urbanisation’)— is part of the ¨remnants of older narratives¨ (Rheinberger, 1994, p. 77) that reveals the reinscription of race science in microbial diversity research as part, I suggest, of the historiality of otherness in human microbiome´s experimental systems. These successive transformations of microbiome science do not correspond with a linear history but, instead, with a ¨patchwork of precocious and deferred actions with its extinctions and reinforcements, interference and intercalations¨ (Rheinberger, 1994, pp. 69–70) in which *race as indigeneity* is concatenated with these scientific undertakings.

### From race science to postgenomics

The concept of race emerged as early as the thirteenth and fourteenth centuries in Europe to refer to ‘lineage, breed, or stock in animals and humans’ (Wade et al., 2014, p. 3). Represented by ‘naturalists’ Carl Linnaeus (1707–1778) and Comte de Buffon (1707–1788), among others, taxonomy developed as part of the Enlightenment project during the eighteenth and nineteenth centuries. It was a way of ordering and classifying plants and human and non-human animals according to their physiological characteristics (Wade et al., 2014, p. 4). The hierarchical taxonomic systems developed at that time, part of what we might call ‘race science’ or ‘raciological science’ (TallBear, 2013) have made their way to our time (El-Haj, 2007). As several social scientists have demonstrated (El-Haj, 2007; Wade et al., 2014), race as a biological concept was not fully abandoned, even after the emblematic UNESCO Statements on Race (1950), a series of documents produced by the United Nations (UN) in the aftermath of the Second World War. As El-Haj argues, the ‘documents did not deny the reality of race as a biological concept’ (2007, p. 286). Instead, the concept of race was gradually substituted with the concept of ‘populations’ (El-Haj, 2007; TallBear, 2013; Wade et al., 2014). In an idiom of ‘percentages and allelic frequencies’, physical traits, the phenotype, were gradually replaced by genetic information, the genotype (Wade et al., 2014, p. 227). The embeddedness of race in population genomics and its molecularisation at the institutional level has been coined as the ‘molecularisation of race’ by Duana Fullwiley (2007) in reference to sociologist Nikolas Rose’s influential notion of ‘molecularisation’ (of the life sciences) (2007).

With the emergence of population genomics in the second half of the twentieth century, the fact that all humans share 99.9 per cent of their genome reached an iconic status. This, in turn, gave rise to several genomic projects aimed at understanding the 0.1 per cent difference among different human populations (via the data mining of their genomes). For example, in 1991 the Human Genome Diversity Project (HGDP) began in Stanford University (US), directed by the influential geneticist Luigi Cavalli-Sforza. The HGDP has established a landmark in population genomic research in terms of biological research on human evolution and migration. The database of the HGDP is in use today (Wade et al., 2014, p. 5). In an epoch of unprecedented environmental damage and extinctions, the HGDP—along with other genomic initiatives such as the International Hapmap Project (2002–2009) or the more recent Human Microbiome Project (2007) and the 1000 Genomes Project (2008–2015)^4^—aims at studying and generating DNA databases of populations of humans and non-humans (especially plants and microbes) before it is too late; before they disappear (Dominguez-Bello, 2013).^5^

In an early and influential article on the use of the bacterium *Helicobacter pylori* as a marker of ancestry and migrations, Dominguez-Bello and Blaser (2011) argue that ‘these microbes [*H. pylori*, ANC] are mostly vertically transmitted, they have evolved within each human group and provide a view of human ancestry’ (Dominguez-Bello & Blaser, 2011, p. 451). They suggest that because ‘human mixing affects microbial phylogeographic signals, and lifestyles impact the human microbiome population structure’, this approach can be useful to gain ‘insights into the population structure of the human microbiome’ (p. 451). Population postgenomics as a tool to unveil multispecies ancestry and establish differences between human populations and their microbial patterns was key for the microbiome scientific community I followed, as a closer analysis to their research outcomes and collaborations shows in the section that follows.

## The microbiomisation of race: the foundations of microbial science´s experimental system

Returning to the results of the MHC research, scientists found major changes in microbial diversity and composition between the two extremes of the urbanisation gradient (i.e., Checherta, hunter-gatherer village, and Manaus, urban city): generally, microbial diversity was at its highest in Checherta and at its lowest in Manaus. The study showed that ‘urbanized spaces uniquely increase the content of human-associated microbes—which could increase transmission of potential pathogens—and decrease exposure to the environmental microbes with which humans have coevolved’ (Ruiz-Calderon et al., 2016, p. 1). The microbial changes documented in the MHC research translate into differences in microbial exposure that might have developmental health implications for humans, more likely ‘immune and metabolic disorders that have become the new disease paradigm in the industrialised world’ (ibid).

In the process of microbiomisation, socio-cultural practices such as cleaning frequency, architecture, family size, along with assessments of age, diet, and kinship are reduced and essentialised to racial categories when microbial species are used as markers of population differences (Núñez Casal, 2019). The comparative microbial genomics population study of the human microbiome in non-Western versus Western populations is a key element of my reformulation of Helmreich´s the ¨microbiomisation of race ¨ as the historiality of otherness in the foundation and condition of possibility of human microbiome science. Often conflated with terms like ¨indigenous¨, ¨nation¨, ¨geographical origin¨ or ¨lifestyles¨, race precedes its signification. Race *is* the foundation of microbial science´s ¨experimental systems¨ as it is already imbricated in the very onset of the research processes. Through the lens of the MHC project, my aim is to show that the ¨microbiomisation of race¨ is constituted by two interlocked movements or phases: (1) the bioprospection of non-Western human and more-than-human microbial biodiversity and (2) the ¨remining¨ (Neimark & Wilson, 2015) of bioprospected data (1) for personalised microbiome profiling online platforms and initiatives.

### Phase 1: bioprospecting biodiversity: restoring the western gut

The comparative study of the human microbiome in non-Western versus Western populations is what constitutes the experimental system of microbial science. It is built on a biodiverse *other* to tackle biodiverse-less *us*. This, unavoidably, is done by bioprospection, a form of piracy or ‘biopiracy’, ‘leading to a loss of power of indigenous people over their own resources’ (Cluis, 2006). In her ethnographic study of bioprospection in Mexico , sociologist of science Cori Hayden points out that bioprospecting ‘is the new name for an old practice: it refers to corporate drug development based on medicinal plants, traditional knowledge, and microbes culled from the “biodiversity-rich” regions of the globe—most of which reside in the so-called developing nations’ (2003, p. 1) (see also Helmreich, 2009; Shiva, 1997).

Bioprospection as the first phase or movement of the ¨microbiomisation of race¨ is a cumulative and particularly endogamous experimental system in which landmark studies such as Dominguez-Bello and Blaser (2011) and Dominguez-Bello´s MHC (2012, 2016) are remined for subsequent similar studies in which the scientists of the original study become part of the successive remining studies. For example, in 2012, Dominguez-Bello took part in a landmark cross-cultural and cross- geographical human microbiome study entitled ‘Human gut microbiota viewed across age and geography’. The aim of the study was to establish the foundations of human genetic and metabolic variation through the characterisation of the human microbiota. The study used faecal samples from three different populations: ‘Amerindians from the Amazonas of Venezuela, residents of rural Malawian communities, and inhabitants of US metropolitan areas’ (Yatsunenko et al., 2012, p. 222). The authors note that:

Pronounced differences in bacterial species assemblages and functional gene repertoires were noted between individuals residing in the USA compared to the other two countries … In addition, the similarity of fecal microbiomes among family members extends across cultures. These findings underscore the need to consider the microbiome when evaluating human development, nutritional needs, physiological variations, and the impact of Westernization (p. 222).

Here, the authors groups human populations into two different categories: one based on race/ethnicity (i.e., ‘Amerindian’) and the other based on nationality/country of residence (residents of the US and residents of Malawi).

As I have previously argued, in the MHC research, the biologisation of the social and cultural is exemplified through the words transculturation, westernisation, and urbanisation. However, published versions of the research avoid invoking social and cultural explanations as well as categorisation into racial/ethnic groups by focusing on differences in the built environments:

Urbanized spaces uniquely increase the content of human-associated microbes— which could increase transmission of potential pathogens—and decrease exposure to the environmental microbes with which humans have coevolved (Ruiz-Calderon et al., 2016, p. 1).

By contrast, drawing on results from the MHC research, several other journal articles co-authored by Dominguez-Bello deliberately focus on nationality and race of the human samples. In an article entitled ‘The microbiome of uncontacted Amerindians’ (Clemente et al., 2015), the authors state that the ‘Yanomani[s] harbor a microbiome with the highest diversity of bacteria and genetic functions ever reported in a human group’ (p. 1). As a result, the article insists on ‘the need for extensive characterisation of the function of the microbiome and resistome in remote non- westernized populations before globalization of modern practices affects potentially beneficial bacteria harbored in the human body’ (p. 6).

In the research article ‘Seasonal cycling in the gut microbiome of the Hadza hunter-gatherers of Tanzania’ (Smits et al., 2017), the authors demonstrate how the Hadza’s human microbiota shifts according to seasonal changes. The study compared the Hadza microbiome profile of 350 stools collected (by Leach) longitudinally over more than a year, with ‘data collected from 18 populations in 16 countries with varied lifestyles’ (p. 802). The results clearly correlate the racial/ethnic category of the Hadza with the Prevotellaceae (bacteria) family and ‘industrialised populations’ (read Western) with the Bacteroidaceae family:

During the cyclic disappearance of taxa, the Hadza microbiota shifts to a state with increased similarity to those of industrialized microbiotas (Fig. S1). Conversely, some OTUs within microbial families common to both traditional and industrialized populations are less seasonally volatile (…). Second, the Prevotellaceae, a member of the Bacteroidetes phylum, is a common family in the Hadza microbiota, leading us to wonder about its relationship to the Bacteroidaceae, a dominant family in industrialized populations, which is also a member of the Bacteroidetes phylum (Smits et al., 2017, p. 804).

It is interesting to note how the designation of social categories of difference varies among different human microbiome studies. While the Tanzania study uses the racial category of the ‘Hadza’ and the socio-economic category of ‘industrialised’, or the study by Yatsunenko et al. (2012) combines racial/ethnic categories (i.e., ‘Amerindians’) with nationality (i.e., US, Malawi), other studies use political categories to signify race/ethnicity. For instance, De Filippo et al. (2010) is a highly cited study on the impact of diet on the gut microbiome that compares ‘European’ children and Burkina Faso children:

BF [Burkina Faso, ANC] children showed a significant enrichment in Bacteroidetes and depletion in Firmicutes (P < 0.001), with a unique abundance of bacteria from the genus Prevotella and Xylanibacter, known to contain a set of bacterial genes for cellulose and xylan hydrolysis, completely lacking in the EU children. In addition, we found significantly more short-chain fatty acids (P < 0.001) in BF than in EU children (2010, p. 14691).

Clearly, comparing populations within a political and economic ‘consortium’ of nation states (i.e., Europe) with a single nation state (i.e. Burkina Faso) is an unequal and problematic comparison. This is accentuated by the fact that neither this study nor the previously mentioned ones provided any explanation about the criteria followed for the categorisation of populations (see also Wade et al., 2014). There is also microbiome literature that uses the term ‘Caucasian’.^6^ In ‘The interpersonal and intrapersonal diversity of human-associated microbiota in key body sites’ (Ursell et al., 2012), co-authored by some of the team I followed, the authors outline the inter- and intrapersonal microbial variation of five body sites across several populations: gut, skin, vagina, mouth, and nose. Summarising the results of the vaginal microbiome, they write:

The vaginal communities of Asian and Caucasian women were most often dominated by lactic-acid producing Lactobacillus than Hispanic and African American women, possibly causing the lower vaginal pH levels found in Asian and Caucasian women (Ursell et al., 2012, p. 1204).

While the terms ‘Asian’ and ‘Hispanic’ are blurry racial/ethnic categories, denoting geographic provenance and colonial history respectively, the word ‘Caucasian’, as the paediatrician Dennis Fortenberry (2013) points out, ‘is a peculiar—but commonly used—racial term because it originates in 18th-century European assumptions of beauty, intelligence, and natural superiority’ (p. 166). In fact, Fortenberry continues, ‘a word steeped in such assumptions amplifies the stigma of sexuality and sexually transmitted infections often associated with racial and ethnic minorities’ (p. 166). Similarly, Wade et al. (2014) argue that mestizaje ‘is a sexualised and gendered practice and ideology’ (p. 19). Genomic research ‘often finds evidence in today’s populations that reflects early colonial mattings between European men and indigenous or African women’ (p. 19). The indissociability and intersectionality of race from other social categories of difference, as Fortenberry and Wade et al. demonstrate, is a remarkable point I have elaborated in relation to class and gender elsewhere (Núñez Casal 2018, 2019, 2021).

There is a characteristic of microbiomisation that is easy to go unnoticed. This has to do with the fact that Western categories of difference are often broader than non-Western ones. Take for example, comparing Burkina Faso children with European children (De Filippo et al., 2010). Or industrialised’ populations versus ‘traditional’ ‘Hadza’ hunter-gatherers (Smits et al., 2017). The Western category is not only broader but is also blurrier. Following a universal and colonial logic, I argue, the Western (microbiome scientists, in this case) defines others (‘hunter-gatherers’, ‘Hispanic’, ‘Amerindians’, and so forth), but does not need to define itself.

Beginning with non-scientific assumptions about cultural and social differences associated with certain populations and geographies (i.e., diet, sanitation, family size, architecture, antibiotic use, child-rearing), microbiome science turns these differences into a heuristic device based on microbial taxonomy. It is then that Tanzanian ‘hunter-gatherers’, ‘Burkina Faso’ children, or simply ‘Hispanics’ have more *Lactobacillus* or *Bacteroidetes* than ‘industrialised populations’ or ‘EU children’. This process involving the biologisation of social groups as pre-existing ‘natural’ phenomena is what I call ‘microbiomisation’. In this sense, microbiomisation entails what philosopher Alfred North Whitehead calls ‘the fallacy of misplaced concreteness’ (1997), also known as the ‘fallacy of reification’, that is ‘the tendency to assume that categories of thought coincide with the obdurate character of the empirical world’ (Duster, 2005, p. 1050).

Postgenomics as inclusion?

Dominguez-Bello is concerned with the ethical implications of her research practices. On the compensation that science should offer to indigenous communities for the mining of their microbiomes, Dominguez-Bello and colleagues suggests that this should be acknowledge in publications scientists should acknowledge in publications while they highlight the importance of empowerment and emancipation for the ‘natives’:

Native peoples must decide their own destinies, but it is our responsibility to provide recognition and safe technologies towards materializing their freedom to choose to remain in their lands, to live their traditional way, and to continue being the guardians of their unspoiled micro- and macro-habitats. *If they do, it will be for the benefit of humanity* (p. 2, my emphasis).

It is worth noting the ambivalence of the discourse. On the one hand, *their* microbiome is a crucial (microbial) ‘reservoir’ for the restoration of *our* own. On the other, on the more ‘ethical’ side, *they* ‘must decide their own destinies’. ‘If *they* do’ choose to keep their ‘traditional’ lifestyles, the benefit will be universal (i.e., ‘humanity’), they argue.

This tension in contemporary biomedical research on populations is often approached from a perspective of inclusion. Medical inclusion, as Steve Epstein shows (2007), is a recent phenomenon. It was during the mid-1980s when reformers pointed out the dangerous flaws of ‘one-size-fits-all’ research (which mostly included white, middle-aged male bodies). The criticism translated into changes in science and pharmaceutical industry policy. While certain aspects of these medical reforms improved disparities in health and disease, ¨by emphasising the biology of difference¨ the inclusiveness of these policy changes and reforms ¨encourage the belief that qualities such as race and gender are biological in their essence¨ along with the ¨mistaken conclusion that social inequalities are best remedied by attending to those biological particularities¨ (Epstein, 2007, p. 11). As Duana Fullwiley has insightfully shown, US race categories are produced and mandated by institutions (e.g., National Institutes of Health) through ¨practices of recruiting, organizing, storing, and comparing human DNA¨ before reaching the lab, unquestionably (2007, p. 22).

These are all important points signalling the biologisation—or, in this case, ‘microbiomisation’— of social categories of difference (race, class and gender) (Núñez Casal, 2019) under frameworks and practices of ‘inclusion’. Furthermore, ‘inclusion’ in postgenomics is not only a policy in science and the pharmaceutical industry, as Epstein (2007) signals, but a societal demand from underrepresented minority groups. sociologist of biomedicine Amy Hinterberger argues that ‘the solution to the dilemmas raised by the unsettled histories of group classification and their increasing entanglement with the futures of genomic medicine is not to stop using categories of differences’ (2012b, p. 220). This is because, among other aspects, measuring and monitoring health disparities would become even more challenging (Epstein, 2007; Hinterberger, 2012b, p. 20).

Some human microbiome research comparing citizens or residents in different countries are clearly designed using the lens of an inclusionary practice of difference (see for instance De Filippo et al., 2010; Yatsunenko et al., 2012). However, in human microbiome science, not all differences and populations belong to a framework of inclusion. This is evident in Dominguez- Bello’s MHC study. Here, the Achuar population of the Peruvian village of Checherta were not selected as participants following an inclusionary practice. The Checherta peoples do not get any medical benefit out of the MHC research, simply because their microbiome is the gold standard for the microbiomes of other populations. Studying their microbiome then is not about inclusion and cannot be explained under that framework. It is rather a question of bioprospection. This is not to say that a scientist like Dominguez-Bello is not well intentioned. The point is that, although Dominguez-Bello and her collaborators provide an ethical framework in which to situate their practice (i.e. bioprospecting biodiversity) (see Dominguez-Bello et al., 2016), the ultimate outcome of the latter is to address a medical problem (i.e. a lack of diversity of microbiomes leading to metabolic, inflammatory, and autoimmune diseases) that affects a specific segment of populations/countries (high-income, fundamentally Western countries). In this sense, knowledge about the microbiome of indigenous communities ‘is evaluated in terms of how well it correlates to orthodox scientific and technological thought, rather than in terms of the belief system that supports it’ (Last & Chavunduka, 1986, p. 217). The comparative population microbiome profiling as part of bioprospecting practices, falls outside a framework of an inclusionary politics of diversity in postgenomic research.

It is evident that, as Hinterberger puts it, ‘the population imagination has not faced in the post-genomic era’ (2012a, p. 76). Yet, in the next section I will show that human microbiome science does not only operate at the level of populations, contradicting what Hinterberger (2012a, 2012b) and other authors (see Fox-Keller, 2010) have argued in relation to genomics and postgenomic medicine. Instead, in my reformulation of the ´microbiomisation of race´, the individualised dimension of human microbiome science, although sustained by microbial DNA data from human populations through bioprospecting practices, gains meaning through informal, online networks of pseudoscientific, commodified, and personalised microbial-related evidence.

### Phase 2. remining bioprospected data: the American Gut Project

Microbiome projects such as MHC are increasingly collaborative and multi-sited. The multi-sitedness of microbiome science is partly conditioned by the varied expertise that interdisciplinary projects like MHC need, and partly because of the maintenance cost of next-generation sequencing technologies that this kind of equipment require. The ‘laboratory is only one of many places’ where human microbiome science ‘accrues value, meaning and relevance’ (Hinterberger, 2012a, p. 72). Assumptions about social categories, microbes, the environment in which microbes reside, the nation state, human and non-human bodies, online platforms, along with DNA, metagenomic data, and High Throughput Sequencing (HTS), constitute this field of research. The scientific configuration of the human microbiome is thereby constituted within a circulation between different research sites and labs, between exchanges of DNA microbial material and gene sequences and the ¨portability¨ and ¨mutability of data that travel¨ (Leonelli, 2015, p. 820).

The microbial DNA samples gathered by Dominguez-Bello and her team in the four locations of the Peruvian and Brazilian Amazon as part of the MHC study were shipped by air to sequence at Knight’s Lab at the University of California in San Diego (US), where they were also remined in the context of the Amerin Gut Project (AGP).

The American Gut Project (AGP) is the first personalised human microbiome initiative co- founded by anthropology-trained entrepreneur Jeff Leach and scientist Rob Knight in 2012. Rob Knight is one of the leading figures of human microbiome research, contributing to over sixty journal articles on microbiome studies per year.^7^ Knight’s Lab is a reference in the field, and not only for researchers like Dominguez-Bello, scientific adviser of the AGP, and Blaser in the context of North American microbiome science.

In an interview, geneticist and influential UK microbiome scientist Tim Spector explained the scale of Knight’s Lab: ‘Rob Knight has set up a very big system, so you can measure 800 samples in one go, which halves the cost of the whole process’ (T. Spector, personal communication, June 29, 2017). Together with Jeff Leach, Spector launched British Gut (BG) in 2015, a subsidiary project of the AGP. All the samples from BG participants are sent to Knight’s Lab to sequence, not because Spector’s department at KCL lacks HTS, but because of its insufficient infrastructure to make the processing of the samples fast and cheap and software to interpret the data (T. Spector, personal communication, June 29, 2017).

The AGP’s purpose is to build a large data set of microbial profiles as well as provide a personalised medicine-like platform in which individual participants (North American) can explore their microbial profile by comparing it with the microbiome of different populations. Between 2012 and 2017, the project amassed the largest open access database in the world of human microbiome samples (> 11,000 ‘citizens’). The majority (94%) of the samples from the 45 countries represented were provided by ‘citizens’ from the UK, US, and Australia. The AGP, as its website claims, has ‘many more samples representing more groups of people than other studies, such as the Human Microbiome Project, Global Gut, or Personal Genome Project’ (AGP, 2018).

**Fig. 3:**
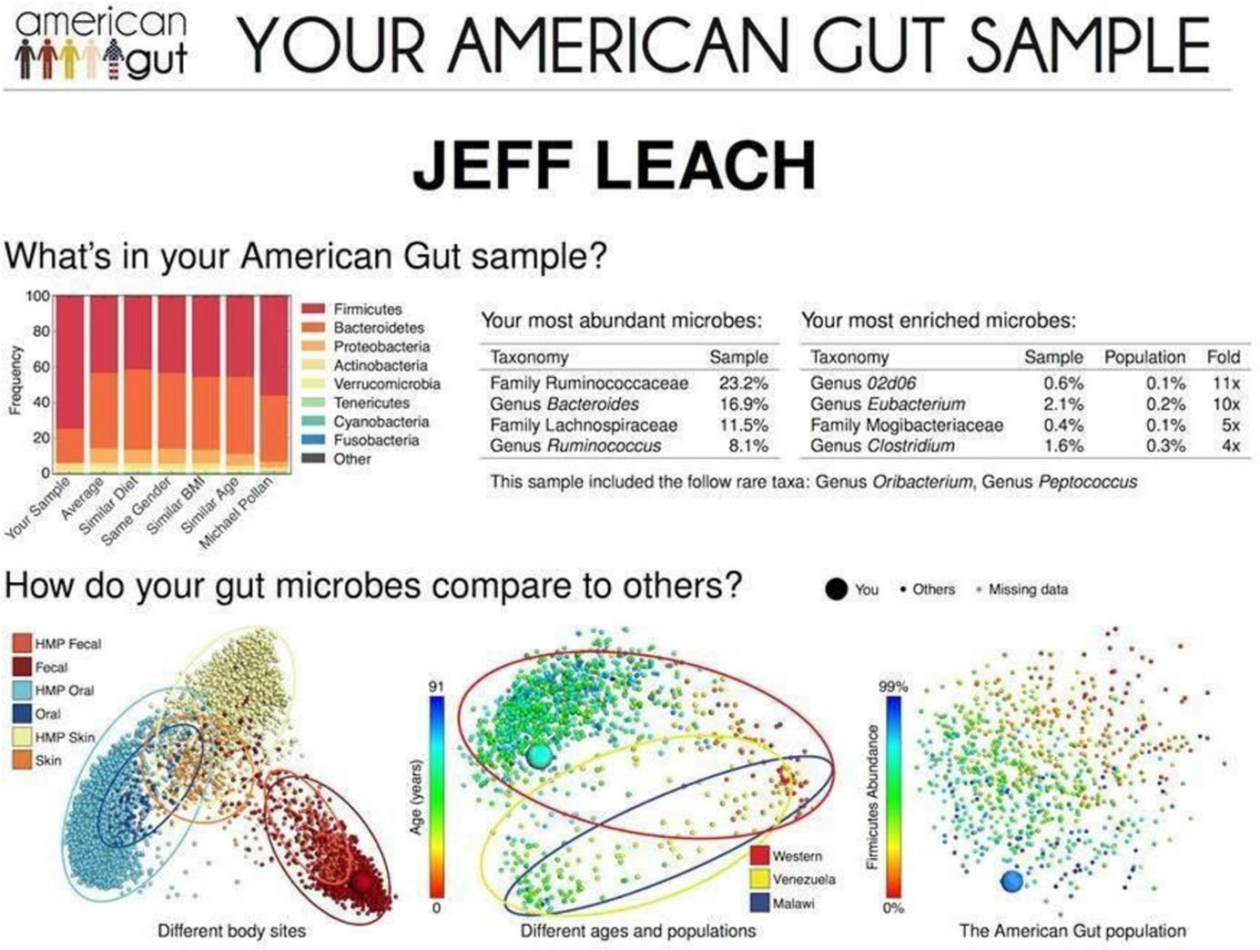
Jeff Leach’s results from his participation in the AGP

Users of the AGP receive a kit for providing samples from the body site(s) of their preference and send the kit back along with a personal survey, detailing their diet and whether they are taking any medication. Once the samples are analysed, they are provided with the results—together with information on how their sample correlates to other profiles, what this data means, and the latest articles and scientific research that relates to their profile (see Fig. 3). Although the initiative was advertised as "open science", thus filling the gap of the US National Institutes of Health's Human Microbiome Project initiative in terms of a very limited number of samples, the so-called AGP "participants" are nevertheless consumers. The cheapest service costs 100 US dollars. In addition to the socio-economic and cultural capital involved in being part of a personalised medicine initiative such as the AGP, the digital skills required for participation cannot be overlooked.

It is important to add that the knowledge and lifestyles needed to biodiversify the microbiome do not only involve consuming initiatives such as AGP. They also involve a healthy diet, exercise, sustained contact with green environments, etc., that is, a series of somatic microbiopolitics (Paxson, 2008; Rose, 2007) which are intrinsically interdependent on social categories of difference such as social class: the higher the socio-economic and cultural capital, the greater the microbial diversity and the lower the susceptibility to inflammatory, autoimmune and metabolic diseases, and vice versa. Such social stratification of microbes and immunities reflects, in turn, the ways in which the intertwinements between economic policies and biomedicine are lived and experienced differently in and by different bodies, intersecting race, class and gender, as I have shown elsewhere (Nuñez Casal, 2018, 2019, 2021a, 2021b, 2024).

The AGP is built upon the remining of microbial data (Benezra, 2016; Delgado, 2021; Neimark & Wilson, 2015). In the AGP, the ´Microbiomes of Homes across Cultures¨ (MHC) project is an indispensable element of the ´microbiomisation of race´s experimental system in order to generate comparative and contextual data for the commodification of personalised microbial profiles. In yet another successive transformation of the research process, the microbial genomic data obtained from population genomic studies such as MHC, as well as from AGP’s participants, is anonymised and added to the database of the Earth Microbiome Project (EMP). The EMP is a massive, open- source and open-access global microbiome study also founded by Rob Knight, whose aim is to catalogue the microbial profiles of the Earth’s ecosystems. This nexus between population genomics data of the human microbiome and an individual microbial profiling acquires meaning through the visualisation of the results participants receive in a PDF file after the samples are processed and sequenced at Knight’s Lab (see Fig. 3).

As occurred with the MHC project, the population categories (i.e., Western, Venezuelan, Malawi) deployed to compare individual samples of the AGP participants are extremely confusing: to what extent is the Venezuelan or the Malawi population ‘traditional’? Does the ‘Western’ category include North American residents or just North American citizens? Many different populations with different lifestyles (that do not fit under the ‘Western’ label) live in the US; how do these categorisations reflect the heterogeneous social (and economic) reality of the US? The AGP does not address any of these questions, nor does it provide any criteria detailing how these categories were established. A closer look at the data and the AGP website reveals that these three (racial) categories of difference based on nationality/residency— ‘Venezuelans’, ‘Malawians’, and ‘Westerners’—are taken from a single human microbiome study conducted by Yatsunenko et al. (in which Dominguez-Bello and Rob Knight are co-authors) in 2012. This cross-cultural and cross-national study is a model for microbiome studies because of the diversity of the variables studied (Fortenberry, 2013). It included mono- and dizygotic twins, children and adults, assessments of residency, kinship, diet, and cultural and social practices and habits. Regarding sample collection, the authors only mention that ‘(s)ubjects were recruited for the present study using procedures approved by Human Studies Committees’ of each of the participating institutions (Yatsunenko et al., 2012, p. 9). Furthermore, there clearly is a remarkable difference between the AGP and the MHC research regarding how the embodied form of the microbial samples is produced in the scientific discourse of microbiome science: the participants of the AGP are ‘citizen scientists’, while the Malawians, Amerindians Venezuelans, and the blurry category of ‘Westerners’ are ‘research subjects’. Clearly, the former have a proactive role: they hold a ‘biological citizenship’ (Petryna, 2002), while the latter are purely passive, devoid of citizenship, ‘illegal’, as it were commodifying the ¨ancestral¨

I situate the AGP as part of a growing wellness industry based on online marketing strategies, in this case, with a focus on individualised microbiome profiling. It consists, for example, of familiarising consumers with medical terms and concepts of the human microbiome through light- hearted language, anecdotes, and strategies for designing and selling products such as nutritional supplements with the claim of macrobiotic properties and enhancements. For example, in 2015, AGP co-founder Jeff Leach, author of several popular books on rewilding (Leach, 2015), launched another small microbiome business associated with the AGP: the Human Food Bar, a nutritional bar sold through the HFP website. ¨Nutrition from the inside out. You are 99% microbiome. It's time you started eating like one. Hadza food¨ (HFP, 2018). These are the slogans printed on the packaging of the bar (Author, 2019). In the AGP, the human microbiome of mostly non- western and indigenous populations is remined and commodified for producing (initially North American) personalised microbial data. In that way, ‘you will know which ancient lineages you have’ (AGP, 2018). Invoking the ancestral and, is a central element of these initiatives (AGP, HFP.). For medical anthropologist Alex Nading, these assertions about the higher microbial diversity of the Hadza in comparison with ‘us “moderns” plays a nostalgic and exclusionary role; It ¨engages with the bodies of colonized others while insisting that they occupy a space beyond “global” environmental or economic life’ (West, 2006, as cited in Nading, 2016, p. 572).

On the assumption of isolation together with using contemporary human and non-human communities and populations as proxy of an ancestral past, microbiologists Graham Rook claimed that the extrapolation of DNA microbial data from African to American or European populations is problematic because these studies overlook evolutionary adaptations to local biologies (Lock & Nguyen, 2010) and, importantly, epigenetic mechanisms. Humans, Rook argues, have developed enormous flexibility through epigenetics. He illustrates this abstract biological idea with a specific case in pregnancy. If a woman with helminths (intestinal parasitic worms) is treated (with an antiparasitic drug) during pregnancy, her baby has a considerably increased likelihood of having allergic disorders, even in communities and populations where allergic diseases are not prevalent. This mechanism is ‘almost certainly epigenetic’ (G. Rook, personal communication, April 21, 2017). This shows, Rooks explains, that helminths protect from developing non-communicable diseases (NCD). Yet, he emphasises that these epigenetic mechanisms also mean that ‘after a few generations in the United States without helminths, helminths are no longer necessary’ and their re-introduction in Western populations would not mean a decrease in NCD.

Rook’s argument contrasts with advocates of biome restoration, which refers to the controlled reintroduction of parasites or bacteria into the human body. DIY biome restoration through helminths and similar therapies such as faecal transplantation are popular among (online) growing communities associated with re-wilding grass-root movements. Contrary to Rook’s argument, geographer Jamie Lorimer argues that helminth therapy implies ‘an ecological model of immunity as involving a multispecies community’ (2016, p. 69) and it offers ‘new ways of thinking companionship and hospitality as more-than-human, but not posthuman, achievements’ (p. 59). I concur with Lorimer in that, contrary to posthumanist hopes of decentring the human (see Hird, 2009; Esposito, 2008, 2011), the ‘human’ of the human microbiome remains the goal of multispecies ethics and therapies. However, I argue that biome restoration through helminths is not about an ‘ecological model of immunity’, as he suggests, but about a delocalised model of immunity based on qualitative, para-ethnographic data (pseudoscientific). Here, the (ancestral) role of helminths in traditional cultures and societies is the principal element sustaining DIY experiments with helminths in the West via an empowered online community. This model of immunity is, in fact, articulated in exclusion (‘us’, moderns, versus others, traditionals) and nostalgia for a (better and healthier) evolutionary past (see TallBear, 2013).

Despite the scientific epistemology of postgenomic microbiome science resting upon a discourse of ‘ecological holism’, co-evolution, and harmonious balance between microbes and humans, through the case of the Microbes of Homes across Culture (MHC) research, along with its online ramifications, the article has demonstrated that microbial science it is not about holism, but about a disembodied knowledge practice based on the expropriation (via bioprospection) of ‘ancestral microorganisms’ and its remining and commodification via associated personalised microbiome science initiatives such as the AGP. While the AGP is does not provide any clinical information, those who can relate to the sequenced microbial DNA are predominantly Western individuals, as human microbiome research is focused on ‘modern diseases’ or ‘lifestyle diseases’ such as diabetes, asthma, and obesity; diseases that affect those populations living in the West or adopting a ‘Western’ lifestyle.

Through the notion of the ‘microbiomisation of race’, I hope to have demonstrated that human microbiome science does not only operate at the level of population as some authors have argued in relation to genomics and postgenomic medicine (Fox-Keller, 2010; Hinterberger, 2012a, 2012b). Instead, the individual dimension of human microbiome science is sustained through the bioprospection and subsequent remining and commodification of microbial DNA data from human populations (mostly non-western). The importance of this argument is paramount as it shows how and to what extent medicalisation, optimisation, and inequalities inhabit newer genomic articulations of difference in microbial science.

## Conclusion

The article has reformulated Stephan Helmreich´s the ¨microbiomisation of race¨ as the historiality of otherness in the foundations of microbial science. Through the lens of the Microbiomes of Homes across Cultures (MHC) project and its associated personalised medicine initiative, the American Gut Project (AGP), I hope to have demonstrated that the ¨microbiomisation of race¨ as a research process of bringing forth, of crystalising or stabilising (Latour, 1988) microbial science, integrates non-scientific assumption about social groups and cultural differences as part of its experimental system. By correlating certain microbial species and diversity and hunter-gatherers, ideas of indigeneity, nation, and ethnicity become microbiomised. Unlike other biological-social interplays—such as the personification of cells, by which biomedicine writes and speaks about cells as if they were interchangeable with persons (Martin, 2006)—in the process of microbiomisation, the ‘social’ (i.e., lifestyle, cultural habits, rituals, traditions, local milieus) is the main element that animates scientific research on microbes.

The ¨microbiomisation of race¨ as the condition of possibility of human microbiome science reveals that its individual dimension is sustained by microbial DNA data from human populations through bioprospecting practices and gains meaning through personalised medicine initiatives, informal online networks of pseudoscientific and commodified microbial-related evidence. Bringing these two mains ´movements´ together, the article has elaborated the notion of the ¨microbiomisation of race¨ as constituted of two interlocking phases: (1) the bioprospection of non-western human and more-than-human microbial biodiversity and (2) the

¨remining¨ (Neimark & Wilson, 2015) of bioprospected data (1) for personalised microbiome profiling online platforms and initiatives. Importantly, while human microbiome science is articulated upon the microbial ‘makeup’ of non-wester(nised) communities, societies, and locales, its results and therapeutics—that is, the health contributions of this biomedical area— are only applicable to medical conditions affecting rich nations (i.e. inflammatory, autoimmune, and metabolic diseases).

In sum, the historiality of otherness in microbial science involves ¨remnants of older narratives¨, specifically the reinscription of race science in postgenomic diversity research (Fullwilley, 2007) as well as ¨fragments of narratives that have not yet been told¨ (Rheinberger, 1994, p. 77), that is, race as the scaffolding upon which human microbiome science´s experimental system rests**.** Through population genomics projects, microbial science reauthorises and reifies race (Benezra, 2020; Helmreich, 2009; Núñez Casal, 2019). Race in the human microbiome science, however, is more than ¨an operational concept¨ with a ¨ghostly presence, one that is there but not there, hiding in shadows and jumping out when least expected¨ (Benezra, 2020, p. 879). While it is often conflated with terms like ¨nation¨, ¨geographical origin¨ or ¨lifestyles¨, it precedes its signification. Race, particularly *race as indigeneity*, is the foundation of microbial science´s ¨experimental systems¨ as it is already imbricated in the very onset of the research processes. Here, the bioprospection of the microbiome of non-Western peoples and territories is much more than a side project or a specific approach within the field: it constitutes the nucleus of its experimental system, opening towards subsequent and cumulative research processes and knowledge production in the field. The spontaneous and unforeseen recurrence of race in microbial postgenomics tells that an ¨experimental system has *more stories* to tell than the experimenter at a given moment is trying to tell with it¨ (Rheinberger, 1994, p. 77).

### Supplementary Information

Below is the link to the electronic supplementary material.

Supplementary file1 (JPG 75 KB)

Supplementary file2 (JPG 60 KB)

## Footnotes

1 Hans-Jörg Rheinberger explains an experimental system as a ¨basic unit of experimental activity combining local, technical, instrumental, institutional, social, and epistemic aspects¨ (1997, p. 238).

2 Anthropologist and popular science writer Jeff Leach’s HFP examines the relationship between diet and microbial diversity. Jeff Leach is also part of Tim Spector’s AGP-affiliated microbiome initiative BG. The difference between the two lies in the provenance of the samples (AGP from North American citizens and/or residents while BG is from British). In 2015, Spector started another microbiome endeavour, MMG, a similar initiative to BG and AGP, although its focus is on gut microbiome analysis and interpretation through accredited health professionals, a service that is neither offered by AGP nor BG.

3 Previously, Knight’s Lab was based at the University of Colorado Boulder, before moving to the University of California, San Diego.

4 The Hapmap Project (2002–2009) was a multinational organisation created to develop a haplotype map (i.e. HapMap) of the human genome, to describe the common patterns of human genetic variation. The 1000 Genomes Project has developed a catalogue of human genetic variation.

5 As it will become evident in the sections that follow, there is an astonishing parallel between the narrative of the MHC project around characterizing the microbiome of "non-westernized populations before globalization" (Clemente et al., 2015) and how Cavalli-Sforza´s HGDP treats the need for get data from isolated populations. This framing of HGDP was one of the reasons for charges of racism and lack of funding (see Reardon, 2005; Saini, 2019). This is an important point that highlights how race is at the core and should raise ethical concerns.

6 For critical engagements on the problems around the term ¨Caucasian¨ in the broader context of biomedicine and population genetics see Popejoy (2021) and Tschirgi et al. (2023).

7 Data from Scopus, 2016
