# Supplementary figures and images for: Race and indigeneity in human microbiome science: microbiomisation and the historiality of otherness
Source: Hist Philos Life Sci. 2024 Apr 2;46(2):17. doi: 10.1007/s40656-024-00614-w (PMC10987353; doi:10.1007/s40656-024-00614-w)

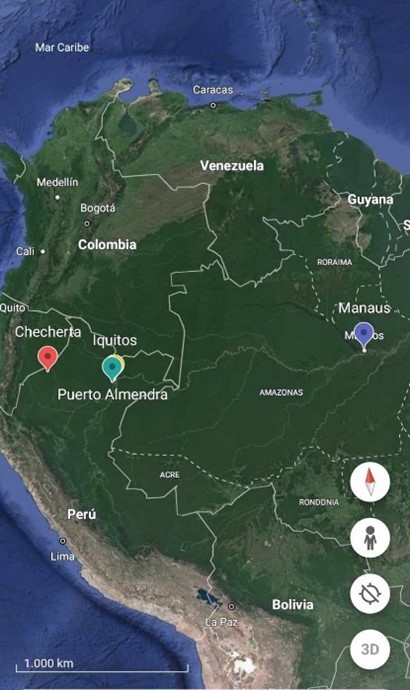

Supplement: Supplementary file 1 — Supplementary file1 (JPG 75 KB) [file 40656_2024_614_MOESM1_ESM.jpg]

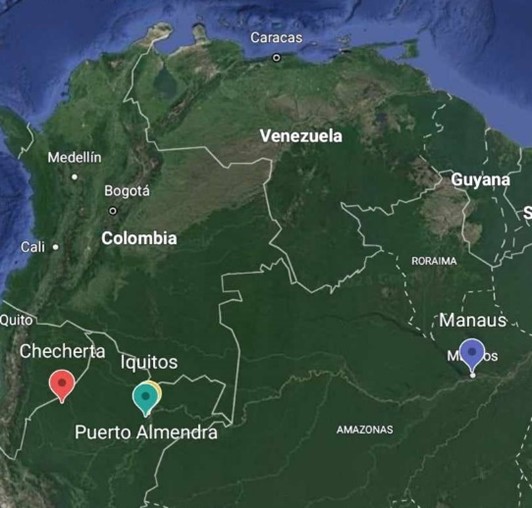

Supplement: Supplementary file 2 — Supplementary file2 (JPG 60 KB) [file 40656_2024_614_MOESM2_ESM.jpg]
